# Peroxisome Deficiency Impairs BDNF Signaling and Memory

**DOI:** 10.3389/fcell.2020.567017

**Published:** 2020-10-14

**Authors:** Yuichi Abe, Yoshiki Nishimura, Kaori Nakamura, Shigehiko Tamura, Masanori Honsho, Hiroshi Udo, Toshihide Yamashita, Yukio Fujiki

**Affiliations:** ^1^Division of Organelle Homeostasis, Medical Institute of Bioregulation, Kyushu University, Fukuoka, Japan; ^2^Faculty of Arts and Science, Kyushu University, Fukuoka, Japan; ^3^Graduate School of Systems Life Sciences, Kyushu University, Fukuoka, Japan; ^4^Institute of Rheological Functions of Food, Fukuoka, Japan; ^5^Department of Molecular Neuroscience, Graduate School of Medicine, Osaka University, Suita, Japan

**Keywords:** peroxisome, Pex2, conditional knockout, memory disturbance, neural stem cell, BDNF, TrkB

## Abstract

Peroxisome is an intracellular organelle that functions in essential metabolic pathways including β-oxidation of very-long-chain fatty acids and biosynthesis of plasmalogens. Peroxisome biogenesis disorders (PBDs) manifest severe dysfunction in multiple organs including central nervous system (CNS), whilst the pathogenic mechanisms are largely unknown. We recently reported that peroxisome-deficient neural cells secrete an increased level of brain-derived neurotrophic factor (BDNF), resulting in the cerebellar malformation. Peroxisomal functions in adulthood brain have been little investigated. To induce the peroxisome deficiency in adulthood brain, we here established tamoxifen-inducible conditional *Pex2*-knockout mouse. Peroxisome deficiency in the conditional *Pex2*-knockout adult mouse brain induces the upregulated expression of BDNF and its inactive receptor TrkB-T1 in hippocampus, which notably results in memory disturbance. Our results suggest that peroxisome deficiency gives rise to the dysfunction of hippocampal circuit via the impaired BDNF signaling.

## Introduction

Peroxisome is an essential subcellular organelle that plays a pivotal role in multiple metabolic pathways, including biosynthesis of ether-phospholipids, β-oxidation of very-long-chain fatty acids (VLCFA), and α-oxidation of branched-chain fatty acids (Wanders and Waterham, 2006). Peroxisomal metabolisms are physiologically consequent as demonstrated by severe pathogenic phenotypes in peroxisome biogenesis disorders (PBDs). PBDs are associated with mutations in *PEX* genes encoding peroxisome biogenesis factors, termed peroxins (Pex). Molecular mechanisms of peroxisome biogenesis have been investigated by several approaches including functional analyses of Pex proteins. Pex2 is a peroxisomal membrane protein containing RING (really interesting new gene) zinc finger at the C-terminal part (Tsukamoto et al., 1991; Fujiki et al., 2006). Pex2 and other peroxisomal RING proteins, Pex10 and Pex12, form RING protein translocation complexes (Miyata and Fujiki, 2005; Okumoto et al., 2014) functioning as the E3 ubiquitin ligase for Pex5, the receptor for the proteins harboring peroxisomal targeting signals (PTS) (Fujiki et al., 2006). Mono-ubiquitination of Pex5 at Cys^11^ is required for the export of Pex5 mediated by the AAA ATPases, Pex1, and Pex6 (Miyata and Fujiki, 2005; Grou et al., 2009; Okumoto et al., 2011). Deficiency of Pex2 results in typical import defects of peroxisomal matrix proteins (Tsukamoto et al., 1991; Shimozawa et al., 1992).

Patients with Zellweger syndrome (ZS), the most severe PBDs, display malformation of central nervous system (CNS) such as disturbance of cortical laminar structure, dysmorphology of Purkinje cells, and dysplasia of inferior olivary nucleus (Volpe and Adams, 1972; de León et al., 1977; Evrard et al., 1978; Steinberg et al., 2006). To date, several *Pex* gene-defective ZS model mice have been generated, showing abnormal development of CNS as observed in ZS patients (Baes et al., 1997; Faust and Hatten, 1997; Maxwell et al., 2003; Abe et al., 2018). Using these ZS model mice, pathological mechanisms underlying PBDs have been studied. We recently reported that *Pex14*^Δ*C*/Δ*C*^ mouse, a ZS model mouse, upregulates brain-derived neurotrophic factor (BDNF) in the inferior olivary nucleus and elevates an inactive truncated form of its receptor, TrkB-T1, in cerebellum (Abe et al., 2018). A combination of the elevated BDNF and the prominent expression of TrkB-T1 in the *Pex14*^Δ*C*/Δ*C*^ mouse gives rise to abnormal morphogenesis of Purkinje cells (Abe et al., 2018). We also showed that cytosolically mislocalized catalase in peroxisome-deficient cells induces the cytosolic reductive state, resulting in the elevation of BDNF (Abe et al., 2020). Taken together, the impairment of BDNF-TrkB signaling pathway might play a causative role in the abnormal CNS development of PBDs. To elucidate peroxisomal functions in the CNS development, CNS-specific *Pex*-knockout mice have also been generated (Hulshagen et al., 2008; Müller et al., 2011). CNS-specific *Pex*-knockout mice demonstrate that peroxisomes in the CNS are essential for the brain development (Krysko et al., 2007; Hulshagen et al., 2008; Müller et al., 2011; Bottelbergs et al., 2012; Rahim et al., 2018). On the other hand, the functions of peroxisomes in the CNS of adult mouse remain largely unknown. The cells aged by replicative senescence are reported to induce the mislocalization of catalase in the cytosol (Legakis et al., 2002; Koepke et al., 2007), suggesting that the aging attenuates peroxisomal biogenesis. Therefore, it is important to unravel the effect of peroxisome deficiency on the functions of adult CNS.

A number of studies reported that BDNF-TrkB signaling in the adult brain is involved in various neural functions such as synaptic plasticity, memory, and neurogenesis (Park and Poo, 2013). Peroxisome deficiency attenuates BDNF signaling, resulting in the malformation of neonatal cerebellum of *Pex14*^Δ*C*/Δ*C*^ mouse at early stages after birth (Abe et al., 2018). In the present study, as a step to investigating the peroxisomal functions in adulthood, we generated tamoxifen-inducible conditional *Pex2*-knockout mouse. Administration of tamoxifen induced the knockout of *Pex2* gene in a whole body as initially assessed in liver, resulting in the defect of peroxisome biogenesis. We analyzed the CNS of conditional *Pex2*-knockout mouse, especially in the hippocampus. Adult mutant mice showed that deficiency of peroxisome biogenesis dysregulates BDNF and TrkB-T1 expressions in hippocampus. Moreover, behavioral experiments revealed that the mutant mouse showed the memory disturbance. Therefore, these results suggest that the peroxisomal function is essential for maintaining the neural integrity in the hippocampus of adult mice.

## Materials and Methods

### Generation of Transgenic Mice

*Pex2*^*flox/flox*^ mice with a floxed exon 7 of *Pex2* gene were generated in Unitech (Kanagawa, Japan). Briefly, genomic DNA corresponding to *Pex2* locus was isolated from bacterial artificial clone (ID: RP23-184F21) containing C57BL/6 genomic DNA. FRT-Neo-FRT-loxP (FNFL) cassette and a loxP site were engineered into flanking exon 7 of *Pex2* gene. The gene targeting vector was as follows: a 2.8-kb short homology arm (11,001–13,800), FNFL cassette, floxed sequence containing exon 7 (13,801–16,607), loxP, and 5.6 kb long homology arm (16,608–22,252) into a vector pBS-DTA carrying the diphtheria toxin A chain (DTA) (Unitech). The replacement vector was linearized by SacII site and electroporated into C57BL/6J embryonic stem (ES) cells that were cultured on G418-resistant mouse embryonic fibroblasts. The resistant colonies were picked up and screened for homologous recombination by Southern blot analysis. Targeted ES cells were injected into C57BL/6 blastocysts to produce chimeric mice. The Neo cassette was then removed *in vivo* by using flp/FRT recombination by crossing Neo-positive offspring from chimeric breeding with transgenic flp *deleter* mice. To generate conditional *Pex2-*knockout mice, *Pex2*^*flox/flox*^ mice were crossed with Cre-ER transgenic mice [B6.Cg-Tg(CAG-cre/Esr1^∗^)5Amc/J] (Hayashi and McMahon, 2002) obtained from Jackson Laboratory. Heterozygous floxed *Pex2* offspring with Cre-ER (*Pex2*^*flox/flox*^/*Cre-ER^+/–^*) were backcrossed to *Pex2*^*flox/flox*^ mice. Homozygous floxed *Pex2* littermates with Cre-ER (*Pex2*^*flox/flox*^/*Cre-ER^+/–^*) and homozygous floxed *Pex2* littermates lacking Cre-ER (*Pex2*^*flox/flox*^) were used as conditional *Pex2*-knockout mice and control mice, respectively. PCR analysis of tail-biopsy genomic DNA was performed using primers 2F (5′-GACTTCTGAGGCACTCATACCTAAC-3′), 2R (5′-ATTGTGAGTTTCAGCATTTCTATGG-3′) to amplify 277 and 435 bp fragments specific for the wild-type allele and targeted allele, respectively. Primers 2F and 2R2 (5′-GACTTCTGAGGCACTCATACCTAAC-3′) were used to amplify 620 bp fragment specific for the disrupted *Pex2* allele. CreF (5′-CGCGATTATCTTCTATATCTTCAGG-3′) and CreR (5′-AGGTAGTTATTCGGATCATCAGCTA-3′) were for 500 bp fragment specific for the Cre gene.

### Induction of Recombination With Tamoxifen

Tamoxifen (Sigma) dissolved at 20 mg/ml in corn oil (Sigma) was orally administrated (10 mg/40 g of body weight) for 5 consecutive days (Hayashi and McMahon, 2002). Two weeks after the last injection, these mice were used for behavioral experiments as described below. Four weeks after the last administration, mouse tissues including brain and liver were excised and homogenized for genotyping, protein analysis, and lipid extraction. Wild-type, *Pex2*^*flox/flox*^/*Cre-ER^–/–^*, and *Pex2*^*flox*/+^/*Cre-ER*^+/–^ mice were used as control mice.

### Contextual Fear Conditioning Test

Apparatus for conditioning and context test consisted of an acrylic square chamber (33 cm × 25 cm × 28 cm) with metal grids (0.2 cm diameter, spaced 0.5 cm apart) covered by white acrylic lid (Udo et al., 2008). Grids were wired to a shock generator to deliver an electric foot-shock as the unconditioned stimulus. On training day, male control and male mutant mice were allowed to freely explore the chamber for 4 min to assess motor function, followed by a 0.2 mA foot-shock. On days 1 and 7, the mice were returned to the same conditioning chamber and freezing behavior was scored to measure contextually conditioned fear without foot-shock. Time spent for freezing and distance traveled were monitored using image J software. Conditioned stimuli (CS) were room and light. Equipment and apparatus were cleaned between trials with 70% ethanol.

### *Pex14*^Δ*C/*Δ*C*^ Mouse

*Pex14*^Δ*C*/Δ*C*^ mouse on a C57BL/6 background was previously described (Abe et al., 2018). Heterozygous offspring (*Pex14*^+/Δ*C*^) were intercrossed to produce homozygous mutant animals. PCR analysis of tail biopsy genomic DNA was undertaken using primers P14F (5′-GTATAAATGTGGGAGTTTCCCTGG-3′) and P14R (5′-GTACTTGTGAACTCTGCTGGTAC-3′) to amplify a 599-bp fragment specific for the wild-type allele and primers P14F and KN52-2 (5′-GTGTTGGGTCGTTTGTTCGG-3′) to amplify a 169 bp fragment specific for the disrupted *Pex14* gene.

### Antibodies and Reagents

Mouse monoclonal antibodies to α-tubulin and nestin (Rat-401) were purchased from BD Biosciences (San Jose, CA) and Invitrogen (Carlsbad, CA), respectively. Mouse monoclonal antibody to 2′,3′-cyclic-nucleotide 3′-phosphodiesterase (CNPase) was from Sigma (St Louis, MO). Rabbit antibodies to BDNF (N-20) and TrkB (H-181) were from Santa Cruz Biotechnology (Texas, CA). We used rabbit antisera to PTS1 peptide (Otera et al., 1998), rat catalase (Tsukamoto et al., 1990), mouse alkyldihydroxyacetonephosphate synthase (ADAPS) (Honsho et al., 2008), C-terminal region of rat Pex2 (amino acid residues 226–305) (Harano et al., 1999), 3-ketoacyl-CoA thiolase (Tsukamoto et al., 1990), acyl-CoA oxidase (AOx) (Tsukamoto et al., 1990), and sterol carrier protein x (SCPx) (Otera et al., 2001). Guinea pig anti-Pex14 antiserum (Itoh and Fujiki, 2006) was also used.

### Lipid Extraction

Total cellular lipids were extracted by the Bligh and Dyer method (Bligh and Dyer, 1959). Briefly, tissue lysates containing 50 μg of total cellular proteins were dissolved in methanol/chloroform/water at 2:1:0.8 (v/v/v) and then 50 pmol of 1-heptadecanoyl-*sn*-glycero-3-phosphocholine (LPC, Avanti Polar Lipids, Alabaster, AL), 1, 2-didodecanoyl-*sn*-glycero-3-phosphocholine (DDPC, Avanti Polar Lipids), and 1, 2-didodecanoyl-*sn*-glycero-3-phosphoethanolamine (DDPE, Avanti Polar Lipids) were added as internal standards. After incubation for 5 min at room temperature, 1 ml each of water and chloroform was added and the samples were then centrifuged at 2,000 rpm for 5 min in Himac CF-16RX (Hitachi Koki, Tokyo, Japan) to collect the lower organic phase. To re-extract lipids from the water phase, 1 ml chloroform was added. The combined organic phase was evaporated under a nitrogen stream and the extracted lipids were dissolved in methanol.

### Liquid Chromatography Coupled With Tandem Mass Spectrometry (LC-MS/MS)

LC-MS/MS analysis of phospholipids was performed as described (Abe et al., 2014) using a 4000 Q-TRAP quadrupole linear ion trap hybrid mass spectrometer (AB Sciex, Foster City, CA) with an ACQUITY UPLC System (Waters, Milford, MA).

### Real-Time RT-PCR

Total RNA was extracted from the tissues using TRIzol reagent (Invitrogen) and first-strand cDNA was synthesized with a PrimeScript RT reagent kit (Takara Bio, Shiga, Japan). Quantitative real-time RT-PCR was performed with SYBR Premix Ex Taq II (Takara Bio) using an Mx3000P QPCR system (Agilent Technologies, Santa Clara, CA). Several sets of primers used are listed in Supplementary Table S1.

### Immunohistochemistry

Mice were deeply anesthetized with isoflurane and then decapitated. The mouse brains were fixed in 4% paraformaldehyde (PFA) in PBS, pH 7.4, for overnight at 4°C and were transferred to 30% sucrose in PBS for 2 days. The brains were embedded in the Tissue-Tek OCT compound (Sakura Finetek Japan, Tokyo, Japan) and subsequently frozen at −80°C. Cryosections were cut at a thickness of 20 μm using a cryostat Microm HM550-OMP (Thermo Fisher Scientific) and were then mounted on MAS-coated glass slides (Matsunami Glass, Osaka, Japan). The sections were permeabilized by ice-cold methanol, blocked by blocking buffer (10% BSA, 0.3% Triton X-100 in PBS) and were then incubated for overnight at 4°C with primary antibody diluted in blocking buffer. After washing with PBS, the sections were incubated with appropriate secondary antibody conjugated to Alexa 488 or 567; for marker staining of nuclei, the sections were incubated with Hoechst 33,242 in PBS for 2 min at room temperature, and then mounted with PermaFluor. For the staining of nestin, anti-nestin antibody was biotinylated by Biotin Labeling Kit (Dojindo, Kumamoto, Japan) according to the manufacturer’s instructions and visualized by FITC-streptavidin (Vector Laboratories Inc., Burlingame, CA). Images were obtained under a LSM710 with Axio Observer.Z1 (Carl Zeiss, Oberkochen, Germany) or AF 6000LX (LEICA, Wetzlar, Germany). Quantitative analysis was performed by Image J software (National Institutes of Health, Bethesda, MD). Quantification of colocalization in randomly selected cells (40∼50 cells) was performed by calculating Pearson’s correlation coefficient using ZEN 2012 software (Carl Zeiss).

### Immunoblotting

Immunoblotting was performed as described (Honsho et al., 2015). Precision Plus Protein All Blue standards (BioRad, Hercules, CA) were used as molecular size markers. Immunoblots were developed with ECL prime reagent (GE healthcare) and detected by LAS-4000 Mini luminescent image analyzer (Fuji Film, Tokyo, Japan). The immunoreactive band intensities were quantified by Image Gauge software (Fuji Film).

### Statistical Analysis

Statistical analysis was performed using R software^1^. All Student’s *t*-tests used were one-tailed. A *P*-value less than 0.05 was considered statistically significant.

### Study Approval

The animal ethics committee of Kyushu University approved all animal experiments.

## Results

### Generation of Tamoxifen-Inducible Conditional *Pex2*-Knockout Mouse

**FIGURE 1:**
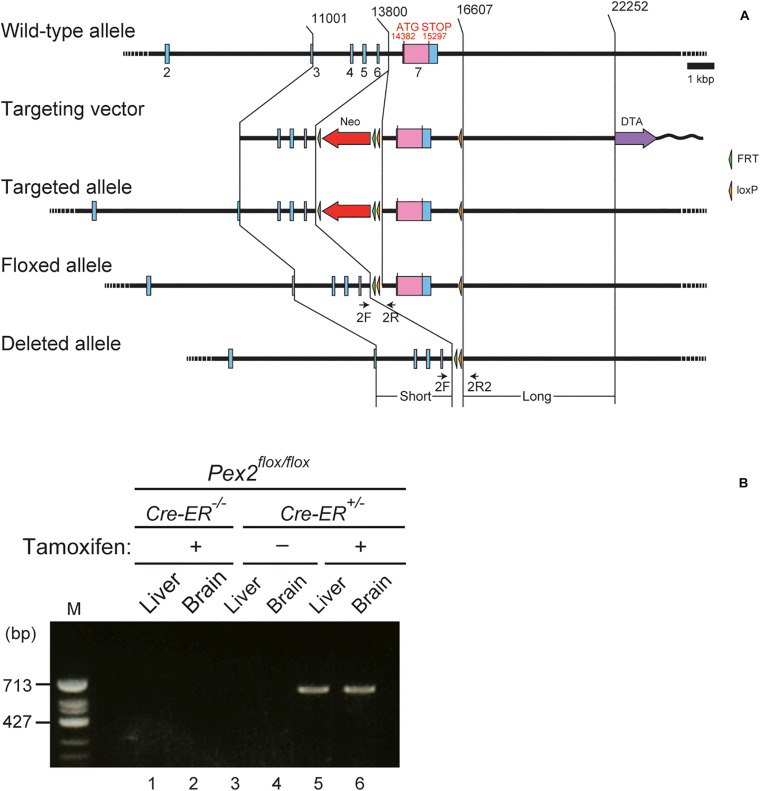
Generation of conditional *Pex2*-knockout mouse. **(A)** Schematic representation of conditional knockout of *Pex2* gene. *Pex2* gene locus (top), targeting vector (second), targeted allele (third), floxed allele (forth), and the *Pex2* exon 7-deleted allele (bottom) were illustrated. Selectable neomycin-resistance (Neo, red arrow) and diphtheria toxin A (DTA, purple arrow) cassettes were shown. Exon sequences were indicated by blue bars and boxes. The coding region of Pex2 protein was indicated by pink box in the exon 7. Primers 2F, 2R, and 2R2 (arrows) were used to assess genomic recombination. **(B)** Genotyping of brain and liver from adult control mouse (*Pex2*^*flox/flox*^/*Cre-ER*^–/–^) and conditional *Pex2*-knockout mouse (*Pex2*^*flox/flox*^/*Cre-ER*^+/–^). PCR products represented Cre-mediated-recombinant gene induced by tamoxifen treatment (620 bp, lane 5 and 6). M, DNA size-markers.

**FIGURE 2:**
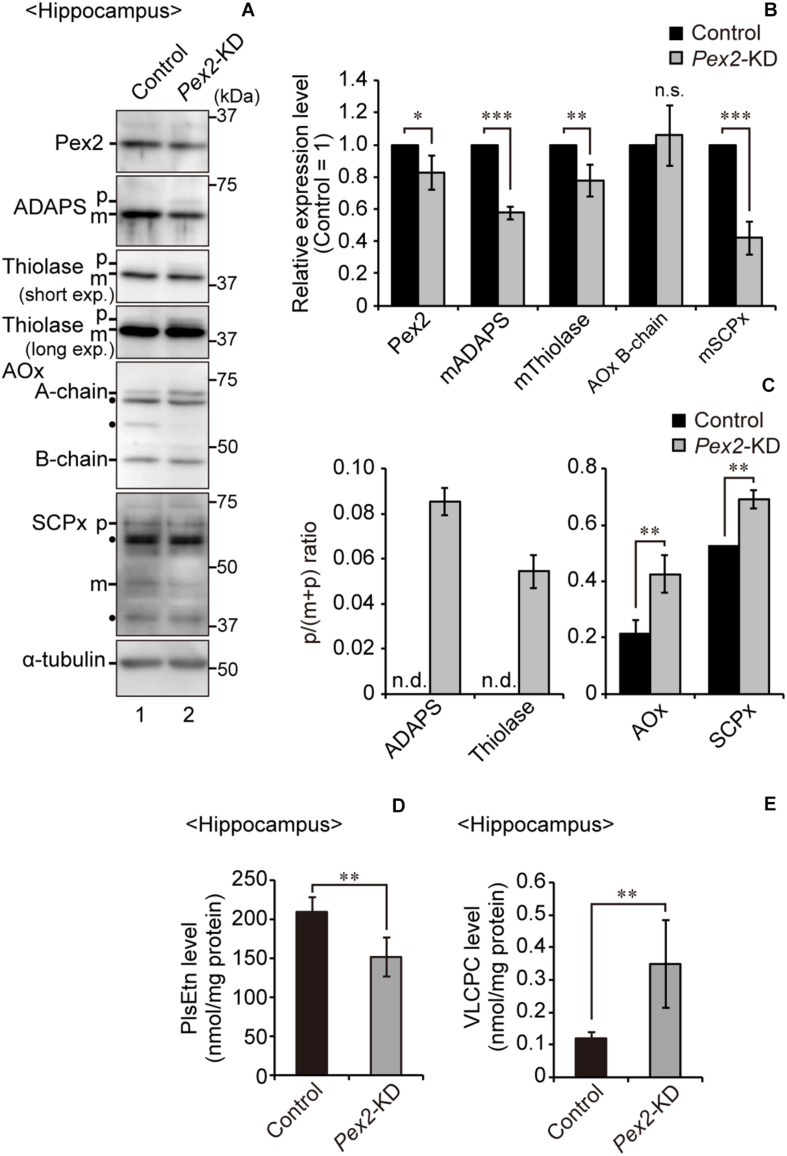
Biochemical analysis of hippocampus of *Pex2*-KD mouse. **(A)** Expression levels of Pex2, ADAPS, thiolase, AOx, SCPx, and α-tubulin in hippocampus of control and *Pex2*-KD mice were analyzed by SDS-PAGE and immunoblotting. Precursor (p) and mature (m) forms of ADAPS, thiolase, and SCPx were as indicated. Conversion of AOx A-chain to B-chain was also assessed. **(B)** The amounts of Pex2 and mature peroxisomal matrix proteins were normalized by α-tubulin level and presented relative to those in control mice (*n* = 3). **(C)** The accumulated precursor proteins including AOx-A chain were shown as p/(p+m) ratio. n.d., not detectable. **(D,E)** Total amounts of plasmalogens **(D)** and VLCPC **(E)** in hippocampus of control and *Pex2*-KD mice were quantified by LC-MS/MS analysis (*n* = 4). Data indicate means ± SEM. *p < 0.05, **p < 0.01, ***p < 0.001, by Student’s *t*-test **(B–E)**.

**FIGURE 3:**
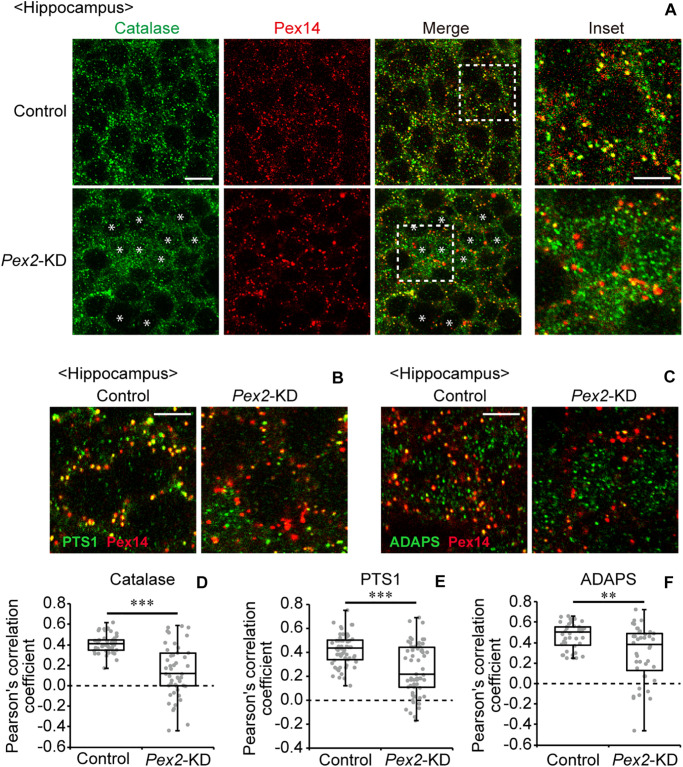
Defect of peroxisomal import of catalase in *Pex2*-KD mouse. **(A)** Coronal hippocampus sections of control and *Pex2*-KD mice were stained with antibodies to catalase (green) and Pex14C (red). Merged views of these two different proteins were also shown (Merge). Asterisks indicate the cells with cytosolically diffused catalase. Scale bar, 10 μm. Higher magnification images of the boxed regions were shown (Inset). Scale bar, 5 μm. **(B,C)** Hippocampus sections were stained with antibodies against PTS1 (**B**, green), ADAPS (**C**, green), and Pex14p (**B,C**, red). Enlarged merged views were shown. Scale bar, 5 μm. **(D–F)** Pearson’s correlation coefficients for colocalization of catalase (**D**, *n* = 50), PTS1 (**E**, *n* = 50), and ADAPS (**F**, *n* = 40) with Pex14 were represented by a set of box plots and dot plots. **p < 0.01; ***p < 0.001, by Mann-Whitney *U*-test.

To investigate the effect of peroxisomal deficiency on the function of CNS in adult mouse, we generated a conditional *Pex2*-knockout mouse using tamoxifen-inducible Cre/*loxP* recombination system (*Pex2*^*flox/flox*^/*Cre-ER^+/–^*; Figure 1). Exon 7 of *Pex2* gene containing the coding region of Pex2 protein was floxed with *loxP* sites (Figure 1A). Administration of tamoxifen gave rise to elimination of exon 7 from *Pex2* gene (Figure 1B, lanes 5 and 6). At 2–3 weeks after the administration of tamoxifen, immunoblotting revealed that Pex2 protein level was partly but significantly decreased in conditional *Pex2*-knockout mouse hippocampus (Figure 2A, lane 2; Figure 2B). We analyzed the mutant mouse at a longer period of time post-tamoxifen administration and did not find any further reduction of Pex2 protein (data not shown). Therefore, we termed it *Pex2*-knockdown (KD) mouse. To assess the peroxisomal matrix protein import, we analyzed the proteolytic processing/conversion of peroxisomal matrix proteins including ADAPS, 3-ketoacyl-CoA thiolase, AOx, and SCPx. The precursors of these proteins including AOx-A chain were elevated in *Pex2*-KD mouse (Figure 2A, lane 2; Figure 2C). Apparently concomitant decrease of mature ADAPS, thiolase, and SCPx was discernible, while AOx B-chain level was not altered (Figure 2A, lane 2; Figure 2B), suggesting that peroxisomal matrix protein import was partially impaired. LC-MS/MS analysis revealed that the reduction of PlsEtn (Figure 2D) and accumulation of VLCFA in PC (Figure 2E), suggesting the attenuation of peroxisomal lipid metabolism. We also performed the immunofluorescent staining of hippocampal sections of *Pex2*-KD mouse using antibodies against peroxisomal matrix proteins. Some neurons showed that catalase was more discernible in the cytosol besides Pex14-positive enlarged peroxisome remnants, so called ghosts (Santos et al., 1988), thereby suggesting that catalase import was attenuated (Figure 3A, asterisks). Moreover, staining with antibodies against PTS1 (Figure 3B) showed defect of matrix PTS1-protein import in hippocampus of *Pex2*-KD mouse. *Pex2*-KD mouse also showed the reduced colocalization of a PTS2 protein ADAPS with Pex14 (Figure 3C). In both of the control and *Pex2*-KD mice, non-specific punctate structures were likely observed around nuclei, as previously reported (Abe et al., 2018). To evaluate the defect of peroxisomal matrix protein import, Pearson’s correlation coefficient was assessed for colocalization of matrix proteins including catalase, PTS1, and ADAPS with Pex14. Pearson’s correlation coefficients in three different stainings were significantly decreased in *Pex2*-KD mouse (Figures 3D–F). Taken together, in hippocampal neurons of *Pex2*-KD mouse, Pex2 is only partially depleted (Figure 2A), giving rise to mosaicked defects in peroxisome biogenesis (Figure 3A).

### Memory Disturbance in *Pex2*-KD Mouse

**FIGURE 4:**
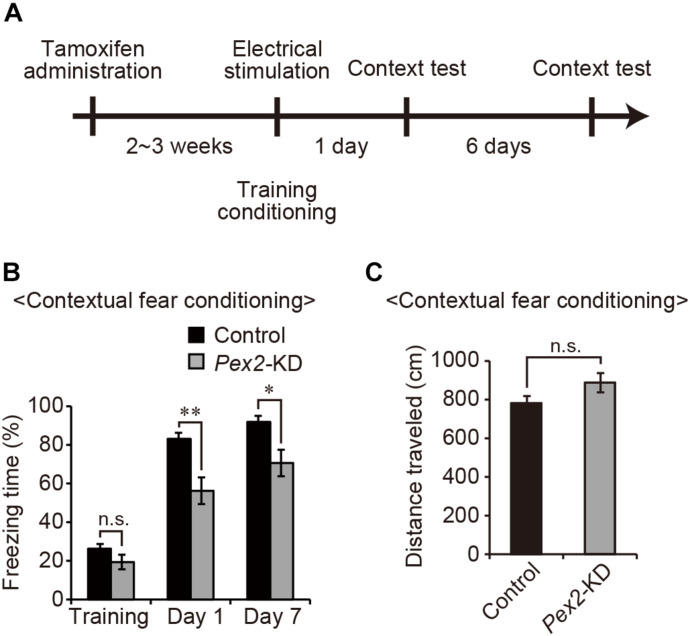
*Pex2*-KD mouse shows memory disturbance. **(A)** Experimental design of contextual fear conditioning test using *Pex2*-KD mice. Two weeks after oral administration of tamoxifen (10 mg/40 g of body weight) for 5 consecutive days, control and *Pex2*-KD mice were placed into a test chamber and were allowed to freely explore the chamber for 4 min (training) followed by foot-shock. On days 1 and 7, the mice were returned to the same conditioning chamber and scored for freezing behavior without foot-shock. **(B,C)** The percentages of time spent in freezing behavior **(B)** and distance traveled **(C)** were represented (control, *n* = 8; *Pex2*-KD, *n* = 10). Data indicate means ± SEM. n.s., not significant, *p < 0.05, **p < 0.01, by Student’s *t*-test **(B,C)**.

Next, to investigate whether peroxisomal abnormality affects memory in *Pex2*-KD mice, contextual fear conditioning test was performed (Figure 4A). On the training day, control and *Pex2*-KD mice were placed in a conditioning box and then received an electric foot-shock as an unconditioned stimulus. On days 1 and 7, *Pex2*-KD mice were frozen, but significantly less than control mice (Figure 4B). However, there was no difference in the distance traveled (Figure 4C), suggesting no deficit in motor function of *Pex2*-KD mouse. Therefore, these results suggested that *Pex2*-KD mice manifest memory disturbance.

### Decrease of Neural Stem Cells in Dentate Gyrus of *Pex2*-KD Mouse

**FIGURE 5:**
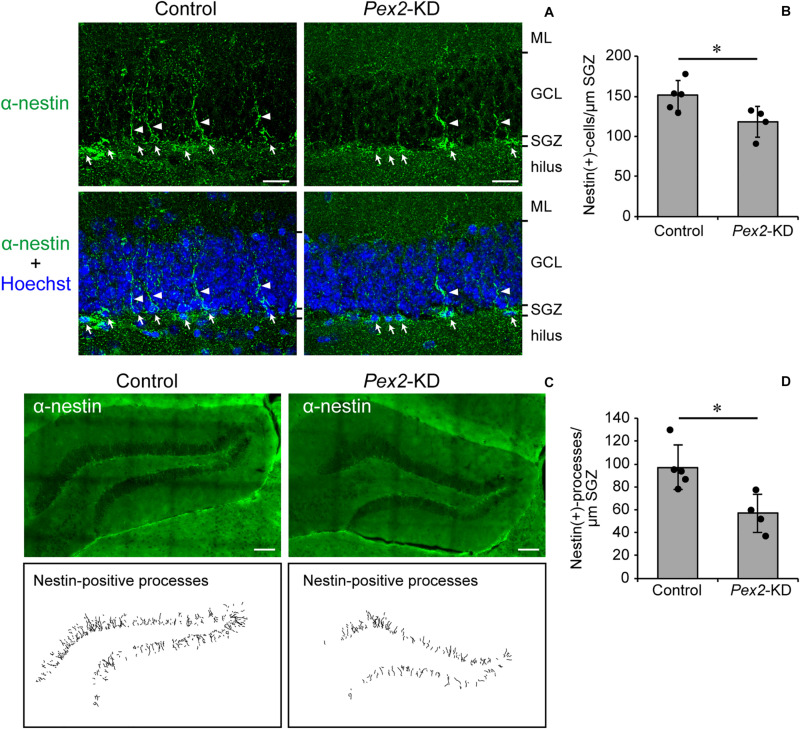
Decrease of neural stem cells in dentate gyrus of *Pex2*-KD mouse. **(A)** Coronal sections of dentate gyrus of hippocampus in control and *Pex2*-KD mice were stained with antibody to nestin (green) and Hoechst 33,242 (blue). Arrows and arrowheads indicate nestin-positive (+) cells and processes, respectively. ML, molecular layer; GCL, granule cell layer; SGZ, subgranular zone. Scale bar, 20 μm. **(B)** The number of nestin-positive (+) cells per 1 -μm SGZ was quantified. **(C)** Dentate gyrus of hippocampus was stained with anti-nestin antibody (upper panels). Traced nestin-positive (+) processes were shown in lower panels. Scale bar, 100 μm. **(D)** The number of nestin-positive (+) processes per 1-μm SGZ was quantified. Data indicate means ± SD. *p < 0.05 by Mann-Whitney’s *U*-test **(B,D)**.

Memory disturbance of *Pex2*-KD mouse suggested the impairment of hippocampal functions. The impairment of cognitive performance in hippocampal-dependent tests, including contextual fear conditioning, is caused by the reduction of neural stem cells (Cameron and Glover, 2015; Gonçalves et al., 2016; Cope and Gould, 2019). We analyzed the neurogenesis in dentate gyrus of adult *Pex2*-KD mouse using anti-nestin antibody. In adult hippocampus, nestin is expressed in type 1 cells, putative neural stem cells possessing radial processes, and type-2a cells, early neural progenitors (Yu et al., 2008; Gonçalves et al., 2016). Nestin-positive cells were aligned (Figure 5A, arrows) along subgranular zone (SGZ) and some cells protruded radial processes (Figure 5A, arrowheads) into granular cell layer (GCL). Statistical analysis revealed that the number of nestin-positive cells per 1 μm SGZ was significantly decreased in *Pex2*-KD mouse (Figure 5B). To evaluate the type 1 cells, nestin-positive processes were traced in the dentate gyrus (Figure 5C). The number of nestin-positive processes per 1-μm SGZ was also significantly reduced in *Pex2*-KD mouse (Figure 5D), suggesting the decrease of neural stem cells. These results suggested that biogenesis and/or functions of peroxisomes were essential for maintaining neural stem cells.

### Peroxisome Deficiency Induces Upregulation of *Bdnf* and *TrkB-T1* in Hippocampus

**FIGURE 6:**
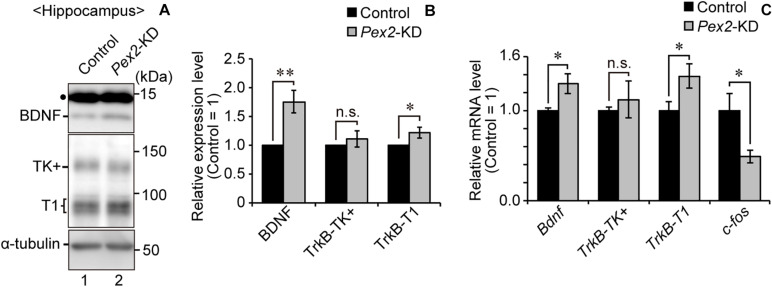
Elevations of BDNF and TrkB-T1 in *Pex2*-KD mouse. **(A)** Expression levels of BDNF, TrkB, and α-tubulin in hippocampus of control and *Pex2*-KD mice were analyzed by SDS-PAGE and immunoblotting. Dot, a non-specific band. **(B)** The amounts of BDNF, TrkB-TK+, and TrkB-T1 were normalized by α-tubulin level and presented relative to those in control mice (*n* = 3). **(C)** mRNA levels of *Bdnf*, *TrkB-TK+*, *TrkB-T1*, and *c-fos* in hippocampus of control and *Pex2*-KD mice were determined by real-time PCR (*n* = 3). Data indicate means ± SEM. n.s., not significant, *p < 0.05, **p < 0.01, by Student’s *t*-test **(B,C)**.

**FIGURE 7:**
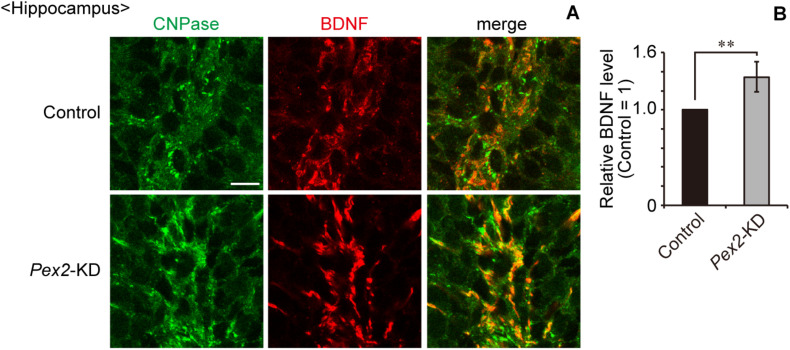
Upregulation of BDNF in hippocampus of *Pex2*-KD mouse. **(A)** Coronal hippocampus sections of control and *Pex2*-KD mice were stained with antibodies to CNPase (green) and BDNF (red). Merged views of the two different proteins were also shown. Scale bar, 10 μm. **(B)** Relative fluorescent intensity of BDNF was quantified (*n* = 4). Data indicate means ± SEM. **p < 0.01 by Student’s *t*-test.

We also assessed the expression level of *Bdnf* in *Pex2*-KD mouse. BDNF protein (Figure 6A, lane 2; Figure 6B) and *Bdnf* mRNA levels (Figure 6C) were significantly elevated in hippocampus of the *Pex2*-KD mouse. Moreover, protein level (Figure 6A, lane 2; Figure 6B) and mRNA level (Figure 6C) of *TrkB-T1* encoding an inactive truncated form of TrkB, but not full-length *TrkB-TK+*, were likewise elevated in hippocampus of *Pex2*-KD mouse. Expression of *c-fos*, a target gene of the BDNF-TrkB signaling pathway (Ip et al., 1993), was significantly reduced (Figure 6C), suggesting the attenuation of BDNF signaling. Immunohistochemical staining and its quantification revealed that BDNF expression was upregulated in hippocampus of the *Pex2*-KD mouse and well merged with CNPase-positive cells, oligodendrocytes (Figures 7A,B).

### BDNF Expression Is Not Altered in Hippocampus of Neonatal *Pex14* Mutant Mouse

Peroxisome-defective *Pex14*^Δ*C*/Δ*C*^ mouse is a ZS model mouse that manifests neonatal death and CNS abnormality such as neuronal migration defect in cortex and cerebellar malformation (Abe et al., 2018). *Pex14*^Δ*C*/Δ*C*^ mouse shows the upregulation of BDNF and the TrkB-T1 in the cerebellum. To investigate whether abnormal expression of BDNF and malformation take place in hippocampus of neonatal *Pex14*^Δ*C*/Δ*C*^ mouse *in vivo*, we analyzed the BDNF and TrkB expression in the hippocampus region at postnatal day 0.5 (P0.5). Immunoblotting showed that hippocampal BDNF expression was not altered in neonatal *Pex14*^Δ*C*/Δ*C*^ mouse (Supplementary Figures S1A,B). Likewise, the expression of TrkB, including TrkB-TK^+^ and TrkB-T1, was not affected in *Pex14*^Δ*C*/Δ*C*^ mouse hippocampus (Supplementary Figures S1A,C). These results suggested that peroxisomes were not involved in the morphogenesis and the regulation of BDNF expression in the hippocampus during fetal development.

## Discussion

The function of peroxisomes in brain of adult mouse has not been examined in details. In this report, we established and analyzed a new transgenic mouse, *Pex2*-KD mouse that shows tamoxifen-inducible defect of peroxisome biogenesis. *Pex2*-KD mouse exhibited memory disturbance and decrease of neural stem cells in hippocampus. BDNF and TrkB-T1 were upregulated in the hippocampus of *Pex2*-KD mouse. Together, these results suggest that the peroxisomal deficiency affects the hippocampal functions in adult mouse.

Bottelbergs et al. (2012) earlier reported the tamoxifen-inducible *Pex5*-KD (*CMV-Tx-Pex5^–/–^*) mouse. *CMV-Tx-Pex5^–/–^* mouse showed motor dysfunction, demyelination, axonal impairment, and microgliosis at 5 or 8 months after tamoxifen administration. In this study, we performed the behavioral experiment with *Pex2*-KD mouse at 2∼3 weeks after the tamoxifen administration. At such early time-points, memory impairment was emerged in *Pex2*-KD mouse (Figure 4B), while the motor function was not affected (Figure 4C). The involvement of peroxisomes in memory function was also suggested by CNS-specific *Pex5* knockout (*Nes-Pex5^–/–^*) mouse that manifests the cognitive impairment (Hulshagen et al., 2008). In *Nes-Pex5^–/–^* mouse, the defect of peroxisome biogenesis in CNS emerges during embryonic day (Krysko et al., 2007). Therefore, memory dysfunction of *Nes-Pex5^–/–^* mouse appears to be owing to the abnormal brain development (Hulshagen et al., 2008; Bottelbergs et al., 2012). On the other hand, CNS function in *Pex2*-KD mouse is intact before the administration of tamoxifen. Thus, the defect of peroxisome biogenesis in *Pex2*-KD mouse induced by administration of tamoxifen apparently impairs the maintenance of neural functions. Taken together, these results suggested that peroxisomal integrity is essential for maintaining the hippocampal memory functions.

It has been reported that inter-organ interaction caused the abnormal brain development in *Pex*-knockout mouse. Liver-specific restoration of peroxisome biogenesis in *Pex5^–/–^* mouse improved the brain morphology (Janssen et al., 2003). Likewise, liver-specific *Pex5*-knockout mouse showed the neuronal migration defect in cortex and malformation of cerebellum (Krysko et al., 2007). Therefore, hepatic peroxisomes likely play a pivotal role in brain development. Further analyses would be required for addressing whether the defect of peroxisome biogenesis in liver affects the neural dysfunction in *Pex2*-KD mouse.

We recently reported that peroxisomal deficiency causes the upregulation of BDNF and TrkB-T1, giving rise to an abnormal morphology of Purkinje cells in the cerebellum of *Pex14*^Δ*C*/Δ*C*^ mouse (Abe et al., 2018). In this study, *Pex2*-KD mouse also showed the elevated levels of BDNF and TrkB-T1 in hippocampus (Figure 6). Our recent study also indicated that cytosolic reductive state caused by cytosolically mislocalized catalase induces the upregulation of BDNF in peroxisome-deficient cells (Abe et al., 2020). However, the upregulation of BDNF in hippocampus was not observed in neonatal *Pex14*^Δ*C*/Δ*C*^ mouse (Supplementary Figure S1). The BDNF expression in hippocampus seems to be differentially regulated in the neonate and adulthood (see Figures 6, 7). The transcriptional regulation of TrkB-T1 in peroxisome-deficient mouse brain also remains to be defined. Further investigations would elucidate the molecular mechanism underlying the up-regulation of BDNF and TrkB-T1 in peroxisome biogenesis-defective neural cells.

BDNF plays an important role in activity-dependent synaptic plasticity such as long-term potentiation in hippocampus (Figurov et al., 1996; Yamada and Nabeshima, 2003). The impairment of BDNF-TrkB signaling pathway in hippocampus is responsible for the defect of learning and memory (Minichiello et al., 1999; Mu et al., 1999). Transgenic mice overexpressing the TrkB-T1 show mild impairment of long-term spatial memory as assessed by water maze test (Saarelainen et al., 2000). In this report, *Pex2*-KD mouse shows the upregulation of BDNF and TrkB-T1 in hippocampus. Taken together, peroxisome deficiency in hippocampus likely induces the attenuation of BDNF-TrkB signaling, resulting in the memory disturbance of *Pex2*-KD mouse. Therefore, peroxisome biogenesis is essential for not only morphogenesis of CNS, but also maintaining the neuronal functions.

Peroxisome biogenesis in *Pex2*-KD mouse hippocampus was mosaically, not completely, impaired as assessed by catalase staining (Figure 3). Nonetheless, the *Pex2*-KD mouse manifests the increased level of BDNF and TrkB-T1 (Figures 6, 7) and the memory disturbance (Figure 4B). These findings suggest that strictly regulated peroxisome biogenesis is required for maintaining the hippocampus-dependent learning. The cells aged by means of replicative senescence show the mislocalized catalase in the cytosol (Legakis et al., 2002; Koepke et al., 2007). Collectively, the less efficient peroxisomal import of catalase caused by senescence most likely gives rise to the dysregulation of BDNF-signaling, resulting in the impairment of hippocampal circuit.

## Data Availability Statement

The raw data supporting the conclusions of this article will be made available by the authors, without undue reservation, to any qualified researcher.

## Ethics Statement

The animal study was reviewed and approved by the Animal Ethics Committee of Kyushu University.

## Author Contributions

YA, ST, MH, TY, and YF conceived and designed the study. YN, KN, ST, and HU generated conditional knockout mice and performed behavioral experiments. YA and YN performed the biochemical and immunohistological analyses of brains. YA, YN, ST, MH, and YF analyzed and interpreted data and wrote the manuscript. All authors contributed to the article and approved the submitted version.

## Conflict of Interest

The authors declare that the research was conducted in the absence of any commercial or financial relationships that could be construed as a potential conflict of interest.

## Footnotes

1 http://www.r-project.org
