# Supplementary material for: Peroxisome Deficiency Impairs BDNF Signaling and Memory
Source: Front Cell Dev Biol. 2020 Oct 14;8:567017. doi: 10.3389/fcell.2020.567017 (PMC7591468; doi:10.3389/fcell.2020.567017)
Supplement: Supplementary file 1 [file Data_Sheet_1.PDF]

Supporting information

Peroxisome deficiency impairs BDNF signaling and memory

Yuichi Abe, Yoshiki Nishimura, Kaori Nakamura, Shigehiko Tamura, Masanori Honsho, Hiroshi Udo, Toshihide Yamashita, and Yukio Fujiki*

*Address correspondence to: Yukio Fujiki, Medical Institute of Bioregulation, Institute of Rheological Functions of Food-Kyushu University Collaboration Program, Kyushu University, 3-1-1 Maidashi, Fukuoka 812-8582, Japan.
E-mail: yfujiki@kyudai.jp; Tel.: +81-92-642-4232; Fax: +81-92-642-4233

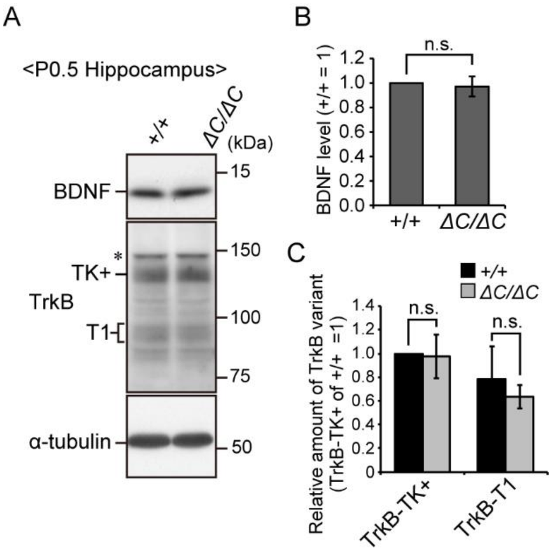

Figure S1. BDNF expression is not altered in hippocampus of *Pex14* mutant mice during early development.

(A) Hippocampus lysates of wild-type (+/+) and *Pex14*$^{\Delta C/\Delta C}$ (*ΔC/ΔC*) mice at P0.5 were analyzed by SDS-PAGE and immunoblotting with antibodies against BDNF, TrkB, and α-tubulin. **(B)** Amount of BDNF was normalized by α-tubulin level and presented relative to that in wild-type mice (n = 5). **(C)** Amounts of two splicing variants of TrkB, TrkB-TK+ and TrkB-T1, were normalized by α-tubulin level and presented relative to those of TrkB-TK+ in wild-type mice (n = 4). n.s., not significant by Student's *t*-test (B and C).

Table S1. Oligonucleotide sequences of primers for real-time RT-PCR

Target mRNA	forward	reverse	product (bps)
Mouse *Bdnf*	5'-gcgcccatgaaagaagtaaa-3'	5'-tcgtcagacctctcgaacct-3'	118
Mouse *TrkB-TK+*	5'-tcttcacctacggcaagcag-3'	5'-tccctgggtgatgcactcta-3'	71
Mouse *TrkB-T1*	5'-tgctcaagttggcgagacat-3'	5'-tccagtgggatcttatgaaacaa-3'	70
Mouse *c-fos*	5'-cgggtttcaacgccgacta-3'	5'-ttggcactagagacggacaga-3'	166
Mouse *Rpl13a*	5'-aacggactcctggtgtgaac-3'	5'-acaggagcagtgcctaagga-3'	118
